# Establishment of a New PNA-FISH Method for *Aspergillus fumigatus* Identification: First Insights for Future Use in Pulmonary Samples

**DOI:** 10.3390/microorganisms8121950

**Published:** 2020-12-09

**Authors:** Laura Cerqueira, Sara Moura, Carina Almeida, Maria João Vieira, Nuno Filipe Azevedo

**Affiliations:** 1 LEPABE—Laboratory for Process Engineering, Environment, Biotechnology and Energy, Faculty of Engineering, University of Porto, Rua Dr. Roberto Frias, 4200-465 Porto, Portugal; nazevedo@fe.up.pt; 2 BIOMODE 2, S.A., Edifício GNRATION, Praça Conde Agrolongo nº 123, 4700-312 Braga, Portugal; uc2018189481@student.uc.pt (S.M.); carina.almeida@iniav.pt (C.A.); 3 Laboratory of Pharmaceutical Chemistry, Faculty of Pharmacy, University of Coimbra, 3000-548 Coimbra, Portugal; 4 Center for Neuroscience and Cell Biology, University of Coimbra, 3004-504 Coimbra, Portugal; 5 INIAV, IP—National Institute for Agrarian and Veterinary Research, Rua dos Lagidos, Lugar da Madalena, 4485-655 Vairão, Vila do Conde, Portugal; 6 CEB—Centre of Biological Engineering, LIBRO—Laboratory of Research in Biofilms Rosário Oliveira, University of Minho, Campus de Gualtar, 4710-057 Braga, Portugal; mjv@deb.uminho.pt

**Keywords:** *Aspergillus fumigatus*, invasive aspergillosis, PNA-FISH, diagnostic methodology

## Abstract

*Aspergillus fumigatus* is the main causative agent of Invasive Aspergillosis. This mold produces conidia that when inhaled by immunocompromized hosts can be deposited in the lungs and germinate, triggering disease. In this paper, the development of a method using peptide nucleic acid-fluorescence *in situ* hybridization (PNA-FISH) is described. The PNA-FISH probe was tested in several strains and a specificity and sensitivity of 100% was obtained. Detection of *A. fumigatus* *sensu stricto* was then achieved in artificial sputum medium (ASM) pre-inoculated with 1 × 10^2^ conidia·mL^−1^–1 × 10^3^ conidia·mL^−1^, after a germination step of 24 h. The PNA-FISH method was evaluated in 24 clinical samples (10 sputum, 8 bronchoalveolar lavage (BAL), and 6 bronchial lavage (BL)) that were inoculated with 1 × 10^4^ conidia·mL^−1^ in sputum; 1 × 10^3^ conidia·mL^−1^ in BL and BAL, for 24 h. Despite a specificity of 100%, the sensitivity was 79%. This relatively low sensitivity can be explained by the fact that hyphae can yield “fungal ball“ clusters, hindering pipetting procedures and subsequent detection, leading to false negative results. Nonetheless, this study showed the potential of the PNA-FISH method for *A. fumigatus* *sensu stricto* detection since it takes only 1 h 30 m to perform the procedure with a pre-enrichment step of 6 h (pure cultures) and 24 h (clinical samples), and might provide a suitable alternative to the existing methods for studies in pure cultures and in clinical settings.

## 1. Introduction

*Aspergillus fumigatus* is a saprophyte filamentous fungus that feeds on decaying organic matter and is able to form a type of spore, named conidium, which can survive in a wide range of aggressive environments and spread through the air [1,2]. Virulence of *A. fumigatus* can be partly explained by thermotolerance, since this fungus grows well at 37 °C but can survive at temperatures over 50 °C [2,3]. Additionally, the small diameter of the conidia (2–3 µm) and their peculiar cell wall composition allows them to travel through the respiratory system towards the pulmonary alveoli, where they can deposit [3,4]. Once there, *A. fumigatus* can cause Invasive Aspergillosis (IA) which, due to the great resistance capacity of this microorganism to antifungal medication [5], has a high mortality rate [6,7]. 

An accurate early diagnosis of *A. fumigatus* in clinical samples such as bronchoalveolar lavage fluid (BAL), sputum and blood, among others, is crucial for a more rational and successful treatment of IA [8,9]. Until now, diagnosis of this microorganism relies on non-specific techniques, such as direct microscopy visualization and serologic tests based on enzyme-linked immunosorbent assay (ELISA) (that target the fungi cell wall components galactomannan and (1,3)-β-D glucan), or on fastidious and time-consuming culture methods [9,10]. PCR-based molecular techniques have also been applied in *A. fumigatus* detection, but a lack of methodological standardization, the occurrence of false positive results and the discrepant results due to the Ct interpretation values are hindering a more widespread use of this technique [9,11]. Fluorescence *in situ* hybridization (FISH) is another molecular-based method that can be applied directly in clinical samples [12], allowing the visual identification of microorganisms and the detection of viable cells with greater certainty, which may decrease the number of false positives.

This technology detects the microorganisms of interest with very high specificity by targeting oligonucleotide probes to specific ribosomal RNA (rRNA), with high copy numbers within cells [13,14]. However, DNA is known to diffuse poorly through the wall of filamentous fungi, even after permeabilization treatments, and as such peptide nucleic acid (PNA) molecules have been used instead as probes to target this group of microorganisms [15]. PNA is a synthetic DNA mimic that has a modified non-charged chemical structure although specific hybridization between the PNA and nucleic acid complementary sequences still occurs according to the Watson-Crick rules [16,17]. Several PNA-FISH methods have already been developed and optimized for a wide range of microorganisms, including bacteria [16,18,19,20,21,22], yeasts [23,24], and filamentous fungi [15,25]. 

In here we consider the development of a PNA-FISH for the specific detection of *A. fumigatus* and testing in clinical samples.

## 2. Materials and Methods

### 2.1. Culture Maintenance and Growth Conditions

All microorganisms used in this study are listed in Table 1. Cell stocks were kept at −80 °C and subcultured once before the experiments. Filamentous fungi strains were maintained in Sabouraud Dextrose Agar (SDA) (Liofilchem s.r.l., Roseto D.A., Italy) or Potato Dextrose Agar (PDA) (Liofilchem s.r.l., Roseto D.A., Italy) for approximately 7 days at room temperature. For each experiment, conidia were harvested by flooding the agar surface with sterilized saline solution containing NaCl 8.00 g·L^−1^ (Sigma-Aldrich, Sintra, Portugal), KCl 0.2 g·L^−1^, Na_2_HPO_4_·2H_2_O 1.44 g·L^−1^, and KH_2_PO_4_ 0.24g·L^−1^ (all from José Manuel Vaz Pereira, Benavente, Portugal) (pH 7.4). Biomass was then suspended in the saline solution with a sterile loop and the final solution collected with a pipette tip to a sterile tube. The heavier fragments were allowed to deposit in the bottom for 5–10 min and subsequently the supernatant was transferred to a new sterile tube [26,27]. *Candida* strains were maintained on Sabouraud dextrose agar for 48 h at 37 °C [28]. Bacterial strains were maintained on Tryptic soy agar (TSA) (Liofilchem s.r.l., Roseto D.A., Italy) for 24 h at 37 °C [29]. Strains were provided by Colección Española de Cultivos Tipo (CECT), Micoteca da Universidade do Minho (MUM), and the American Type Culture Collection (ATCC).

### 2.2. Design of a PNA Probe for the Specific Detection of A. fumigatus

In order to find a specific *A. fumigatus sensu stricto* sequence to be used as a target for the PNA probe, 18S and 28S rRNA sequences were evaluated. From these, twenty four 28S rRNA gene sequences available at the National Center for Biotechnology Information (NCBI) (http://www.ncbi.nlm.nih.gov) and SILVA (http://www.arb-silva.de/browser/) databases were selected. The final dataset comprised 11 *A. fumigatus*, 6 *Penicillium* sp., 4 *Aspergillus terreus* and 3 *Neosartorya fischeri* sequences. Regions of interest were selected using ClustalW (European Bioinformatics Institute; http://www.ebi.ac.uk/clustalw/) and putative probe sequences were then ranked according to highest specificity and sensitivity towards *A. fumigatus*. High GC percentage and no self-complementary structures in the probe were also considered [30]. ProbeCheck (http://www.arb-silva.de/fish-probes/probe-design/) was then used to assess theoretical specificity and sensitivity. Specificity was calculated as nAfs/(TnAf) × 100, where nAfs stands for the number of non-*Aspergillus fumigatus* strains that did not react with the probe and TnAf is the total of non-*Aspergillus fumigatus* strains examined. Sensitivity was calculated as Afs/(TAfs) × 100, where Afs stands for the number of *Aspergillus fumigatus* strains detected by the probe and TAfs is the total number of *Aspergillus fumigatus* strains present in the databases [30,31].

The selected sequence was then synthesized (Panagene, Daejeon, Korea). The N terminus of the oligomer was connected to Alexa Fluor 594 via a double AminoEthoxyEthoxy Acetyl linker (AEEA) linker.

### 2.3. Hybridization Conditions

Conidial suspensions were first centrifuged (10 min; 10,000× *g*), to remove any agar residues from scraping the culture plates, and resuspended in the saline solution prepared as described above. Subsequently, 1 × 10^6^ conidia·mL^−1^ of that suspension were resuspended in peptone-yeast extract-glucose (PYG) containing peptone 1 g·L^−1^ (Merck, Darmstadt, Germany), yeast extract 1 g·L^−1^ (Merck), and glucose 3 g·L^−1^ (Liofilchem s.r.l., Roseto D.A., Italy) (pH 5) [32] or Potato Dextrose Broth (PDB) (Liofilchem s.r.l., Roseto D.A., Italy). Conidia germination was achieved overnight (for approximately 16 h) at 37 °C and 120 rpm. Suspensions were then centrifuged for 10 min, 10,000× *g*, and the obtained supernatant replaced by saline solution. This washing was repeated twice. The subsequent hybridization on glass slides was assessed as previously described [16,19,30], with some modifications. In short, the suspensions were dispensed in 8 mm well slides (Marienfeld, Lauda-Königshofen, Germany) and then allowed to air dry. For permeabilization and fixation of *A. fumigatus*, 30 µL of 4% paraformaldehyde (*w/v*) followed by 50% ethanol (*v/v*), for 10 min each, were dispensed in the wells with care to cover the entire surface. Slides were then allowed to air dry. Approximately 20 µL of hybridization solution, which contained 200 nM of the specific probe, 10% (*w/v*) dextran sulphate, 10 mM NaCl, 30% (*v/v*) formamide, 0.1% (*w/v*) sodium pyrophosphate, 0.2% (*w/v*) polyvinylpyrrolidone, 0.2% (*w/v*) ficoll, 5 mM disodium EDTA, 0.1% (*v/v*) Triton X-100, and 50 mM Tris-HCl (all from Sigma-Aldrich, Sintra, Portugal, except disodium EDTA that was from Pronalab, Lisbon, Portugal), was then added. The slides were covered with coverslips and incubated for the different times and temperatures under study. Afterwards, coverslips were carefully removed and the slides were transferred to a Coplin jar containing prewarmed washing solution, that consisted of 5 mM Tris Base, 15 mM NaCl, and 1% (*v/v*) Triton X-100 (all from Sigma-Aldrich, Sintra, Portugal). The washing step was carried out for 30 min at the same temperature as hybridization. The slides were allowed to air dry, mounted with one drop of mounting oil, and covered with a coverslip. Several temperatures (53 °C, 55 °C, 58 °C and 60 °C) and times of hybridization (30, 45, 60, 90 min) were studied for signal-to-noise ratio assessment. Non-inoculated samples were also prepared as negative controls. All slides were stored in the dark and visualized under the microscope in less than 24 h.

After optimization of the hybridization conditions, the probe was applied in smears of 8 *A. fumigatus*, 12 *Aspergillus* non-*fumigatus*, and other 13 related species or typical infectious agents found in pulmonary diseases (Table 1). 

### 2.4. Germination Assays Using the Selected Probe

For inoculation and germination assays, the same protocol described for optimization of hybridization conditions was used. Briefly, conidial suspensions were rinsed by centrifugation twice and resuspended in saline solution. A concentration of 1 × 10^6^ conidia·mL^−1^ of that suspension was resuspended in PYG (37 °C, 120 rpm) allowing conidia germination [26,32]. At selected time points (0 h, 2 h, 4 h, 6 h, 8 h, 12 h), 1 mL of solution was centrifuged for 10 min and 10,000× *g*, after which the supernatant was replaced by saline solution. This procedure was repeated two times to remove any residue of the growing media and then hybridization in the glass slides was performed.

### 2.5. Detection of A. fumigatus in Artificial Sputum Media (ASM)

A test with artificial sputum media (ASM) was used to assess the baseline PNA-FISH conditions to be used in the clinical matrixes.

After inoculation with different concentrations of MUM 07.05, ATCC 46645, CECT 20366, and CECT 2071, ranging from 1 × 10^1^ to 1 × 10^4^ conidia·mL^−1^ of sample, 1 mL of ASM composed of 5 g mucin from porcine stomach, 4 g DNA, 5.9 mg diethylenetriamine pentaacetic acid (DTPA), 5 g NaCl, 5 g aminoacids (all from Sigma-Aldrich, Sintra, Portugal), 2.2 g KCl (José Manuel Vaz Pereira, Lisboa, Portugal), and 5 mL egg yolk emulsion (Oxoid, ProBiológica, Portugal) per one liter of distilled water (pH 7.0) [33] was added to BACTEC^TM^ Plus Aerobic/F Medium (Becton Dickinson bottles, Quilaban, Portugal) and incubated at 37 °C, 120 rpm. At specific times (6 h, 8 h, 12 h and 24 h), samples of 1 mL were recovered from each culture to perform hybridization on glass slides, as described above.

### 2.6. PNA-FISH Method Testing in Clinical Samples 

Several patients from Centro Hospitalar do Médio Ave, E.P.E (CHME) (Vila Nova de Famalicão, Portugal), who collected samples from sputum, bronchial lavage (BL), or bronchoalveolar lavage (BAL), participated in this study (February to May 2017), with a total of 60 samples collected. The study was previously approved by the hospital ethics committee, and informed consent was obtained from all patients. Part of the samples was used for optimization tests of the germination step and of the PNA-FISH procedure and 24 (10 sputum, 6 BL and 8 BAL) were used for the final validation test.

Before proceeding to the validation test on real samples, a preliminary assay was done to evaluate the PNA-FISH performance in the previously optimized conditions. Nine clinical samples (3 Sputum, 3 BAL and 3 BL), negative by culture, were artificially inoculated with MUM 07.05 *A. fumigatus* strain. Although the concentration used in BAL and BL was the same as that estimated in the ASM experiment (1 × 10^3^ conidia·mL^−1^), for sputum a higher concentration was used (1 × 10^4^ conidia·mL^−1^), as it was estimated in preliminary tests (data not shown). To increase the chances of reducing the protocol time, lower times of incubation were also tested (8 h and 12 h). In the case of sputum, and after inoculation, 0,1 g of *N*-acetyl-L-cysteine (Merck) was added to 1 mL of sample for 20 min (without shaking) followed by 1 mL of phosphate-buffered saline (PBS). Subsequently, the samples were added to 30 mL of the selected medium and incubated at 37 °C, 120 rpm. At each time point (8, 12, and 24 h), a sample of 1 mL was recovered from each culture and was centrifuged for 10 min at 10,000× *g* and resuspended in distilled water. This step was repeated two more times to remove culture medium residues. Hybridization was performed as previously described. All clinical samples were cultured as a control and were submitted to mycological examination. The samples were plated on SDA plates and were incubated at 37 °C for 7 days. Then, *A. fumigatus* was identified using standard macroscopic morphologic criteria, such as color, size, and texture of colony.

### 2.7. Blind Study: Testing of PNA-FISH in Clinical Samples

Twenty-four clinical samples (10 Sputum, 6 BL and 8 BAL) were tested blindly by PNA-FISH to assess diagnostic performance in the detection of *A. fumigatus sensu stricto*. Of these 24 samples, 13 samples (1 mL) were inoculated blindly with the lowest concentration (strain MUM 07.05) that was determined in the previous experiments (1 × 10^4^ conidia·mL^−1^ for Sputum and 1 × 10^3^ conidia·mL^−1^ for BL and BAL). In the case of sputum, after inoculation, 0,1 g of *N*-acetyl-L-cysteine (Merck) was added to 1 mL of sample for 20 min (without shaking) followed by 1mL of PBS. The germination procedure was then performed as described above until 24 h, where a 1 mL sample was taken and centrifuged twice (10 min, 10,000× *g*) and resuspended in distilled water, and the hybridization was performed as described.

At the same time, 100 μL of each sample were used to assess the number of colony-forming units (CFUs), either directly or after 1:10 dilutions in SDA plates. The plates were incubated at room temperature for 5–6 days before counting.

### 2.8. Microscopy Visualization

Cells were analyzed using two epifluorescence microscopes: BX51 Olympus, (Hamburg, Germany) equipped with a CCD camera (DP71; Olympus) and Nikon Eclipse 80i (Japan) with a camera Nikon DS-Fi1 and software NIS-Elements B.R. 3.2 (Izasa, Japan), both with a filter (TRITC) sensitive to the fluorochrome molecule attached to the PNA probe (excitation, 530 to 550 nm; barrier, 570 nm; emission long-pass filter, 591 nm). Visualization of negative controls was assessed with the other filters present in the microscopes that are not sensitive to the probe fluorescence signal.

### 2.9. Statistical Analysis

Statistical validity parameters specificity and sensitivity and respective 95% confidence intervals (CIs) were determined using the VassarStats: Website for Statistical Computation (http://vassarstats.net).

## 3. Results

### 3.1. Theoretical Specificity and Sensitivity Determination

The identification of possible sequences was made comparing 28S rRNA *A. fumigatus sensu stricto* sequences with other related species. The selected sequence, with the highest number of *A. fumigatus* sequences detected and the lowest number of non-*A. fumigatus* sequences detected, was 5′-ACAGAGCAGGTGACA-3′. The sequence targeted the 28S rRNA between positions 274 and 288 of the *A. fumigatus* A1163 (Accession number ABDB01000088; SILVA database), and was therefore named FUM274. The probe lacked self-complementarity and presented 53% GC content. After the selection of the specific probe for the detection of *A. fumigatus* its theoretical specificity and sensitivity were determined using the LSU database of the ProbeCheck program. The search showed that FUM274 detected 79 out of 80 *A. fumigatus* 28S sequences available in the database that cover the alignment position of the selected probe and therefore the theoretical sensitivity was calculated as 98.8% (95% CI, 92.4–99.9). No other species presented sequences complementary to the probe, and as such specificity was 100% (95% CI, 94.2–100).

### 3.2. PNA-FISH Performance

Different temperatures and hybridization times were studied to evaluate which were the optimal conditions for the probe to work. The best performance in terms of strongest signal-to-noise ratio was achieved at 55 °C and 1 h of hybridization. For the other times tested, only minimal differences in probe performance were observed. As expected, for the optimized hybridization conditions, the probe only hybridized with *A. fumigatus* strains (Table 1). Therefore, 100% specificity (95% CI, 87–100) and 100% sensitivity (95% CI, 59.8–100) for the probe was obtained. 

**Figure 1:**
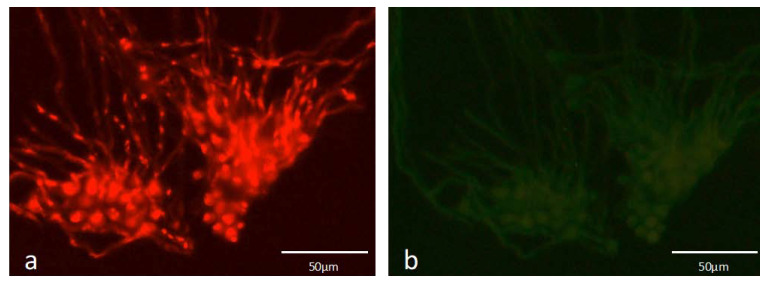
Epifluorescence microscope visualization of *A. fumigatus* ATCC 46645 after overnight growth for germination, in peptone-yeast extract-glucose (PYG) medium with FUM274 (**a**). Visualization of the same microscopic field at the green channel (negative control of FUM274) (**b**). Images were obtained with equal exposure times.

A preliminary test using *A. fumigatus* conidia without a pre-germination step and overnight grown hyphae was performed to see if the probe could hybridize to different fungal structures in the same manner. It was observed that FUM274 only presented an easily observable fluorescence signal in germinated cells (Figure 1).

**Figure 2:**
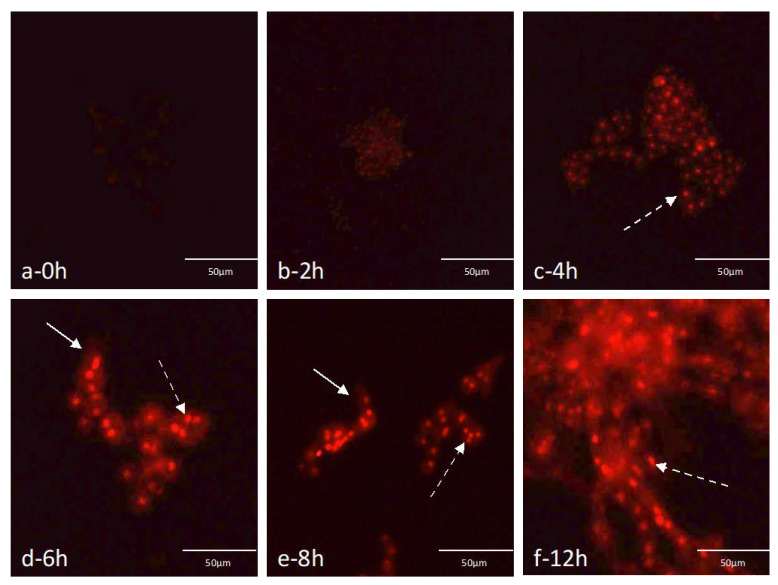
Epifluorescence microscope visualization of *A. fumigatus* MUM 07.05 germination along time, in PYG medium with FUM274. A very faint fluorescence signal can be observed at 0 h (**a**); signal was still weak at 2 h and 4 h (**b**,**c**); At 6 h, 8 h, and 12 h the signal intensity increases substantially (**d**–**f**). It can be noticed that conidia starts swelling at 4 h, and that partial germination occurs at 6 h and 8 h (filled arrows), whereas full germination is achieved at 12 h. In certain locations inside the cells the fluorescence signal is more intense (dotted arrows). All images were obtained with an equal exposure time.

Because a minimization of the germination time was desirable to increase the speed of the PNA-FISH test, the hybridization performance in different developmental states of *A. fumigatus* (from resting conidia until full germination) was monitored (Figure 2). It was possible to qualitatively evaluate signal-to-noise ratio according to the different steps of conidia germination. Conidia started to swell after only 2 h, but this event was more evident after 4 h. Nonetheless, for both times the fluorescence signal was faint. Partial germination was observed at 6 h and 8 h, when apical growth of hyphae occurs. At this point, fluorescence was much brighter, extending up to 12 h where full germination occurred. Because 6 h, in general, was the time when the fluorescence signal-to-noise ratio started to be stronger, in the subsequent experiments this time was selected as the first pre-germination step time used before undertaking the hybridization with FUM274. The fluorescence signal intensity was not uniform inside the cells with very bright spots clearly visible (dotted arrows; Figure 2). All *A. fumigatus* used in this study were tested, and the same results were obtained.

### 3.3. Detection of A. fumigatus in Artificial Sputum Medium Contaminated Samples 

In artificially contaminated samples, four *A. fumigatus* strains (MUM 07.05, ATCC 46645, CECT 20366, and CECT 2071) were tested (Table 2). When applied on ASM the probe could only detect fully germinated *A. fumigatus* at 24 h. However, an initial concentration of 1 × 10^2^ to 1 × 10^3^ conidia·mL^−1^ was needed for the detection of the different strains. Intermediate time points (16 h and 20 h) were also monitored for the MUM 07.05 and CECT 20366 strains in ASM, and positive hybridization was observed (data not shown). 

### 3.4. Optimization Assays Using Clinical Samples

From all clinical samples obtained, only 2 (1 sputum, 1 BL) were positive in culture testing. Although this result was expected by the low occurrence of fungal infections [12,34], the low percentage of positive samples would imply that the number of samples needed to obtain a meaningful specificity, and the sensitivity value for PNA-FISH would be very high. For that reason, negative samples were randomly artificially contaminated. 

Before proceeding to the final validation test on real samples, preliminary assays were performed to evaluate the PNA-FISH performance in the previously optimized conditions. Nine samples (3 sputum, 3 BLs, and 3 BALs) were inoculated using the minimal concentration previously assessed. Different enrichment time points were tested (8 h, 12 h, 24 h). The number of positive results increased with enrichment time (Table 3) with the best results obtained at 24 h of incubation, confirming the previous results. Nonetheless, at this time point some hyphae aggregates were observed, which hampered the pipetting process and hinder the subsequent microscopic observation.

Although *A. fumigatus* was positive by culture in all samples, at 8 h it was only detected in one BL and one BAL sample by PNA-FISH. Even though conidia swelling and germ tubes were observed, it was only possible to see a few fungal structures per sample at the microscope. At 12 h, three samples (one sputum and 2 BALs) gave negative results. However, at 24 h, was possible to observe fluorescent signal in all samples, the results being consistent with the culture method.

### 3.5. Blind Study

In order to validate the PNA-FISH method, a blind test with 24 samples (10 sputum, 6 BL, and 8 BAL) using the conditions (time and inoculum concentration) previously optimized was performed. From these 24 samples, 13 samples were contaminated with the lowest cell concentration (1 × 10^3^ conidia·mL^−1^ in BAL and BL; 1 × 10^4^ conidia·mL^−1^ in sputum), using MUM07.05 strain and tested for 24 h germination.

Of the thirteen contaminated samples, 3 gave false negative results, one in each kind of matrix (Table 4). At 24 h, the observed development of fungal aggregates in the liquid medium hindered the process of pipetting the required volumes. As a control, all the samples were cultured and the results were in agreement with the inoculation scheme.

The total sensitivity and specificity for the method for 24 h of germination were 79% (CI 95%, 49–94%) and 100% (CI 95%, 66–100%) respectively.

## 4. Discussion

An early diagnostic of invasive fungal infections is of high importance since it can lead to a more effective treatment [8,9,35]. At the moment, detection of *A. fumigatus* is based on PCR techniques, culturing methods, or serologic tests. However, and as described above, all these methods present drawbacks that can lead to an inaccurate *A. fumigatus* identification [9,10,11]. This work intends to give insights about the development of a PNA-FISH method that can be provided as a suitable alternative for the detection of this microorganism. For a start, the PNA-FISH procedure is simple and fast to perform and can be applied directly on a wide range of samples without the requirement for DNA isolation, manipulation, and amplification [16,18,19,20,21,22,23,25]. Furthermore, for an accurate diagnosis, specific evidence of hyphae in clinical samples such as blood, sputum, BAL, or in tissues is required [12]. PNA-FISH is the only molecular method that allows for the visual inspection of the samples and may hence provide additional information about the *A. fumigatus* germinating stage and cellular integrity. 

While not tested against other *Aspergillus* species from the section *Fumigati*, for the optimized hybridization conditions, the selected 28S rRNA PNA probe is able to distinguish *A. fumigatus sensu stricto* from other *Aspergillus* sections, namely *A. ibericus*, *A. ochraceus*, *A. clavatus*, *A. versicolor*, *A. terreus*, *A. tubingensis*, *A. oryzae*, *A. flavus* and *A. niger*, with very high specificity and sensitivity [36], although changes in fluorescence signal were observed for different germination stages. These differences in signal intensity may be attributed to the increased levels of rRNA content of the microbial cells as they germinate and to the different cell wall composition and structure. The number of ribosomes inside the cell influences the hybridization signal because the FUM274 targets the 28S rRNA of *A. fumigatus*. In fact, earlier studies have shown that, if all other parameters are kept constant, the relationship between signal intensity and ribosome number is linear [37]. For *A. fumigatus*, new genetic material and proteins are required when primary germ tube formation starts [38]. This leads to a high rate of ribosome production, which has been found to increase ten times from the passage of dormant cells to when the germination begins [39]. The experiments in pure cultures have shown an intensity increase of the fluorescence signal in the moment that coincides with the beginning of the germination stage (mostly 6 h), which points to a clear influence of the ribosomal concentration. Furthermore, in fungi the rRNA genesis starts at the nucleolus and mature rRNA subunits are exported to cytoplasm [38], and they can either be “free ribosomes” or incorporated in the rough endoplasmic reticulum, a cellular organelle placed near the nucleus. This high activity in the nucleolus can explain the fact that we have observed specific locations of brighter fluorescence spots concentrated near the cell nucleus in every germination stages. In some cells it was possible to observe more than one fluorescence spot (Figure 2), which might also be explained by the fact that cells are undergoing a mitotic/duplication cycle generating daughter cells [40]. In fact, it has been noticed that subapiccal cells in hyphae may contain three to four equally spaced nuclei [41].

Cell wall composition and structure might also affect hybridization performance, as the FUM274 needs to diffuse through this barrier before reaching the cytoplasm. In the resting conidia cell wall there is a layer of hydrophobic proteins, called hydrophobins, protecting the cell. The disintegration of this layer marks the end of the cell dormancy, and only then the cells start swelling and the primary germ tubes emerge to form hyphae [42,43]. While the choice for a PNA probe has been made in part to minimize issues related to probe diffusion [31], the relevance of this factor in explaining our changes in the fluorescence signal may not be discarded. The PNA-FISH method developed here is hence more robust once the *A. fumigatus* start to germinate. For pure cultures, strains can be distinguished as early as 6 h. Different times were observed when the method was applied to ASM, where the best pre-germination step was found to be 24 h. However, since it was possible to detect cells at 16 h and 20 h, an overnight growth is likely to be enough for a successful hybridization. The same behavior seemed to occur in real clinical samples. Before the final test, a preliminary assay with nine clinical samples was performed to evaluate the PNA-FISH performance in the optimized conditions. Thus, the clinical samples were inoculated with the minimum concentration previously determined and they were let to germinate during 8 h, 12 h, and 24 h. At the first time point, *A. fumigatus* was hard to detect, and this may not only be due to the fact of conidia start swelling and generating the germ tube later in the natural sample, but also due to samples composition. These clinical samples are known to be very complex matrixes composed by different cells, proteins, and other molecules [44,45] that can mask the presence of smaller conidia cells within the cellular milieu. Nevertheless, at 12 h enrichment, *A. fumigatus* was not detected in three contaminated samples using PNA-FISH, with longer enrichment time (24 h) all contaminated samples were positive for *A. fumigatus* using the PNA-FISH method. In this assay, the culture method was performed as control and positive result for *A. fumigatus* were achieved in all samples, at 8 h, 12 h, and 24 h corroborating the evidence that the fungus germination phase influences the PNA-FISH method performance. 

In the final blind test, despite a relative specificity of 100% being obtained, only 79% sensitivity was achieved. At 24 h, hyphae formed clusters like “fungal balls” after their full germination and, consequently, it became more difficult to pipette and detect the *A. fumigatus*, causing false negative results. This fungal agglomeration can be due to the cell wall modifications. During germination, the α(1,3)-glucans become exposed at the cell surface of swollen conidia [46]. Due to their physical properties, chains of α(1,3)-glucans interact between themselves and are responsible for the aggregation of swollen conidia. In order to reduce the pre-germination step (<24 h) in clinical samples and eliminate the possible aggregate clusters, the incorporation of an intermediate sonication step must be considered in the future. Alternatively, glass beads to disintegrate the molecular structures can also be used [47]. Even though, without any treatment, the FUM274 detection limit threshold (1 × 10^2^–1 × 10^4^ conidia·mL^−1^) is similar to the real-time PCR method described earlier [48,49]. Future work will be carried out in order to improve the PNA-FISH detection limit. Compared to culture that takes about 7 days for definite fungal identification, PNA-FISH can be considered a rapid and reliable method since it takes only 1 h 30 m to perform the procedure, with a pre-enrichment step of 6 h (pure cultures) or 24 h (clinical samples).

## 5. Conclusions

In this paper, a new molecular diagnostic method is proposed using a specific PNA probe for direct visualization of *A. fumigatus sensu stricto* by FISH. The FUM274 proved to be capable to detect this specific fungus, in a minimum time of approximately 6 h for pure cultures and 24 h in diverse clinical samples, which clearly shortens the culture time-to-result, allowing the results to be obtained about 5 days earlier, improving patient treatment and clinical guidelines. It was observed that several factors can influence the enrichment phase, and further tests must be perform in order to decrease the procedure time and detection limit, improving sensitivity. Afterwards, tests on non-inoculated clinical samples must also be performed in order to perceive the real ability of this method to detect *A. fumigatus* in this kind of sample. Nevertheless, the results obtained have shown that the PNA-FISH method holds great promise to be an alternative to the unspecific and fastidious traditional methods currently used for *A. fumigatus* diagnosis.

## Figures and Tables

**Table 1:** List of strains used in this study, together with the results obtained with the peptide nucleic acid-fluorescence in situ hybridization (PNA-FISH) probe specificity/sensitivity test. CECT, Colección Española de Cultivos Tipo; MUM, Micoteca da Universidade do Minho; ATCC, American Type Culture Collection. (+)—positive fluorescent result by PNA-FISH; (−)—negative fluorescent result by PNA-FISH.

Strains Tested	PNA−FISH Outcome
*Aspergillus fumigatus* MUM 02.24	+
*Aspergillus fumigatus* MUM 07.05	+
*Aspergillus fumigatus* MUM 9802	+
*Aspergillus fumigatus* ATCC 46645	+
*Aspergillus fumigatus* CECT 2071	+
*Aspergillus fumigatus* CECT 20190	+
*Aspergillus fumigatus* CECT 20228	+
*Aspergillus fumigatus* CECT 20366	+
*Aspergillus ibericus* MUM 03.49	−
*Aspergillus ochraceus* MUM 9302	−
*Aspergillus clavatus* MUM 9717	−
*Aspergillus versicolor* MUM 00.20	−
*Aspergillus terreus* MUM 9409	−
*Aspergillus tubingensis* MUM 06.152	−
*Aspergillus oryzae* MUM 10242	−
*Aspergillus flavus* MUM 00.06	−
*Aspergillus flavus* MUM 9201	−
*Aspergillus niger* MUM 92.13	−
*Aspergillus niger* MUM 01.01	−
*Emericella nidulans* var. *echinulata* MUM 9832	−
*Neosartorya fisheri* var. *glabra* MUM 9836	−
*Penicillium brevicompactum* MUM 02.12	−
*Penicillium chrysogenum* MUM 061.70	−
*Mucor hiemalis* MUM 9732	−
*Trichoderma viride* MUM 9754	−
*Candida parapsilosis* ATCC 22019	−
*Candida tropicalis* ATCC 750	−
*Candida glabrata* ATCC 2001	−
*Candida albicans* ATCC 1472	−
*Pseudomonas aeruginosa* PAO1	−
*Pseudomonas aeruginosa* CECT 111	−
*Escherichia coli* K12	−
*Staphylococcus aureus* CECT 239	−

**Table 2:** Results of *A. fumigatus* detection in sheep blood and in artificial sputum medium (ASM) for the different times sampled. No positive results were obtained for concentrations lower than 1 × 10^2^ conidias·mL^−1^. (+)—positive fluorescent result by PNA-FISH; (−)—negative fluorescent result by PNA-FISH.

	ASM			
Concentration (conidia·mL^−1^)	6 h	8 h	12 h	24 h
**MUM 07.05**				
1 × 10^4^	−	−	−	+
1 × 10^3^	−	−	−	+
1 × 10^2^	−	−	−	−
1 × 10^1^	−	−	−	−
**ATCC 46645**				
1 × 10^4^	−	−	−	+
1 × 10^3^	−	−	−	+
1 × 10^2^	−	−	−	+
1 × 10^1^	−	−	−	−
**CECT 20366**				
1 × 10^4^	−	−	−	+
1 × 10^3^	−	−	−	+
1 × 10^2^	−	−	−	+
1 × 10^1^	−	−	−	−
**CECT 2071**				
1 × 10^4^	−	−	−	+
1 × 10^3^	−	−	−	+
1 × 10^2^	−	−	−	+
1 × 10^1^	−	−	−	−

**Table 3:** PNA-FISH results regarding *A. fumigatus* detection in nine artificially contaminated clinical samples (1 × 10^4^ conidia·mL^−1^ sputum; 1 × 10^3^ conidia·mL^−1^ BL and BAL) for 8, 12, and 24 h. (+)—positive fluorescent result by PNA-FISH; (−)—negative fluorescent result by PNA-FISH.

Sample	Culture	PNA−FISH Outcome		
		8 h	12 h	24 h
**Sputum**				
1	+	−	+	+
2	+	−	−	+
3	+	−	+	+
**BL**				
1	+	−	+	+
2	+	−	+	+
3	+	+	+	+
**BAL**				
1	+	−	−	+
2	+	−	−	+
3	+	+	+	+

**Table 4:** PNA-FISH blind test results. Thirteen clinical samples were inoculated artificially with the minimum concentration (1 × 10^4^ conidia·mL^−1^ in sputum; 1 × 10^3^ conidia·mL^−1^ in BL and BAL). (+)—positive fluorescent result by PNA-FISH; (−)—negative fluorescent result by PNA-FISH.

Sample	Inoculation	Culture	PNA−FISH Outcome
**BAL**			
1	+	+	+
2	+	+	+
3	−	−	−
4	+	+	+
5	−	−	−
6	+	+	+
7	+	+	−
8	−	−	−
**BL**			
9	−	−	−
10	+	+	+
11	−	−	−
12	+	+	+
13	−	−	−
14	+	+	−
**Sputum**			
15	+	+	+
16	−	−	−
17	+	+	+
18	+	+	−
19	+	+	+
20	−	−	−
21	+	+	+
22	+	+	+
23	−	−	−
24	−	−	−
